# Insights into the structure and function of the RNA ligase RtcB

**DOI:** 10.1007/s00018-023-05001-5

**Published:** 2023-11-07

**Authors:** Matthieu Moncan, Hassan Rakhsh-Khorshid, Leif A. Eriksson, Afshin Samali, Adrienne M. Gorman

**Affiliations:** 1 Apoptosis Research Centre, University of Galway, Galway, Ireland; 2 School of Biological and Chemical Sciences, University of Galway, Galway, Ireland; 3 Department of Chemistry and Molecular Biology, University of Gothenburg, 405 30 Göteborg, Sweden; 4 CÚRAM SFI Research Centre for Medical Devices, University of Galway, Galway, Ireland; 5 Biomedical Sciences, Upper Newcastle, University of Galway, Galway, H91 W2TY Ireland

**Keywords:** 3′–5′ RNA ligase, Archease, Cancer, Crystal structure, RNA 2′,3′-cyclic phosphate and 5′-OH ligase [RtcB], RNA ligation, Transfer RNA [tRNA], tRNA ligase complex, Unfolded protein response [UPR], X-box-binding protein 1 [XBP1]

## Abstract

To be functional, some RNAs require a processing step involving splicing events. Each splicing event necessitates an RNA ligation step. RNA ligation is a process that can be achieved with various intermediaries such as self-catalysing RNAs, 5′–3′ and 3′–5′ RNA ligases. While several types of RNA ligation mechanisms occur in human, RtcB is the only 3′–5′ RNA ligase identified in human cells to date. RtcB RNA ligation activity is well known to be essential for the splicing of XBP1, an essential transcription factor of the unfolded protein response; as well as for the maturation of specific intron-containing tRNAs. As such, RtcB is a core factor in protein synthesis and homeostasis. Taking advantage of the high homology between RtcB orthologues in archaea, bacteria and eukaryotes, this review will provide an introduction to the structure of RtcB and the mechanism of 3′–5′ RNA ligation. This analysis is followed by a description of the mechanisms regulating RtcB activity and localisation, its known partners and its various functions from bacteria to human with a specific focus on human cancer.

## Introduction

Early discoveries concerning splicing of RNA molecules guided the curiosity of researchers towards identifying RNA ligases and ligation mechanisms that are responsible for the maturation of RNA species, such as mRNAs and tRNAs, that contain introns. Although some RNA ligation processes are performed by self-catalysing RNAs, other processes are dependent on protein enzymes. There are only two identified proteins that catalyse RNA ligation in human cells, namely RtcB (also known as HSPC117, C22ORF28), which catalyses 3′–5′ RNA ligation and C12orf29, which catalyses 5′–3′ RNA ligation and was identified very recently [1]. This review will focus on RtcB.

RtcB enzymes are responsible for 3′–5′ RNA ligation in archaea, bacteria, algae and animals [2]. The substrates of RtcB are two RNA fragments, one phosphorylated at the 3′-end and the other hydroxylated at the 5′-end. In animals, the ligase activity of RtcB is required for maturation of intron-containing tRNAs and is, thus, essential for general protein translation. In addition to its role in the splicing of tRNAs, RtcB re-ligates exons of *XBP1* mRNA after removal of a 26-nucleotide intron [3–5]. This non-conventional splicing of *XBP1* mRNA begins with endoribonuclease activity of IRE1α (hereafter named IRE1), an endoplasmic reticulum (ER) transmembrane protein that is activated as part of the unfolded protein response [6, 7]. *XBP1s* (‘s’ for spliced) mRNA encodes a transcription factor that enhances transcription of itself, as well as several genes involved in the unfolded protein response and lipid synthesis [8–11]. While these two RNA substrates are the main ones known in human, the downstream functions of RtcB ligase activity may not be restricted to these two pathways, as its ligase activity may be generalised to any compatible 3′- and 5′ RNA-ends.

Here, we review the structure of human RtcB and how the enzyme catalyses 3′–5′ RNA ligation. As data for human RtcB are relatively scant, we rely on the high levels of inter-species homology between RtcB enzymes to learn from studies performed in other organisms. We also discuss recent research regarding regulation of RtcB. Finally, as relatively little is known regarding the role of RtcB in pathological conditions, we discuss what can be learned about the various roles of RtcB from bacteria to human cells and how this information reveals the potential and challenges of RtcB as a therapeutic target for human cancer.

## RtcB activity and interacting partners

### Mechanism of catalysis

**Fig. 1:**
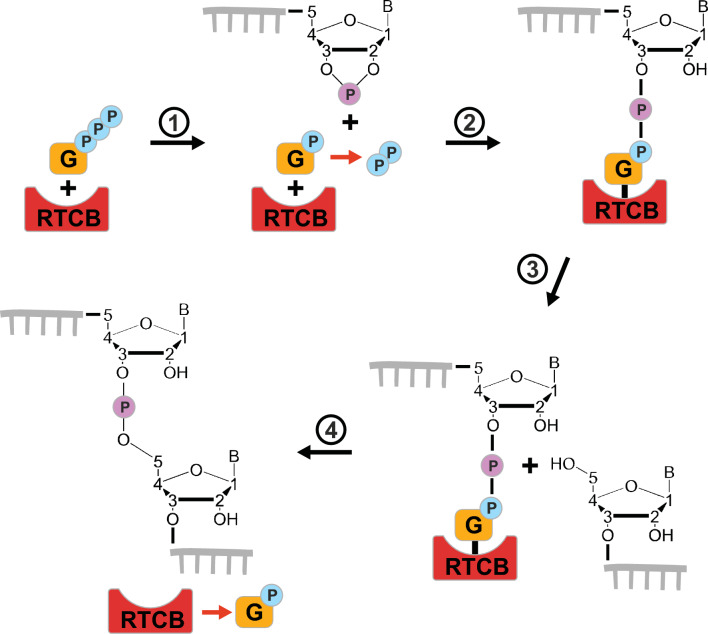
RtcB 3′–5′ RNA ligation mechanism. (1) RtcB hydrolyses GTP to form a covalent bond with GMP. (2) RtcB-GMP hydrolyses the 2′–3′-cyclic phosphate of the 3′-end of the RNA substrate, generating a 3′-phosphate-GMP-RtcB intermediate. (3 and 4) The 5′-OH end of the RNA substrate attacks the 3′-phosphate end to form a phosphodiester bond ligating both RNA-ends and provoking the release of GMP from RtcB. Although not represented in this schematic, binding of GMP to RtcB requires two divalent metal ions. While 2′–3′-cyclic phosphate is the main substrate for RtcB RNA ligation, 3′-phosphate ends can also be ligated although with a reduced efficiency

Most of what is known about the mechanism of ligation by RtcB comes from studies on its prokaryotic paralogues that catalyse a 3′–5′ RNA ligation reaction, joining two RNA fragments while hydrolysing GTP. The phosphate group in the newly formed phosphodiester bond originates from one of the RNA fragments, where it is bound to both 2′- and 3′-carbons of ribose in a cyclic manner at the terminus of RNA fragment (2′, 3′-cyclic phosphate) (Fig. 1). The second RNA fragment has a hydroxyl group attached to its free 5′-carbon at the terminus (5′-OH). In the first step of the ligation reaction, RtcB reacts with GTP and hydrolyses it to GMP, to which it remains covalently bound through a conserved histidine. Then, an RtcB-GMP-3′-phosphate intermediate is generated by hydrolysis of the 2′–3′-cyclic phosphate end to produce a 3′-phosphate end. Finally, the 5′-OH end of the second RNA substrate attacks the 3′-phosphate end of the first RNA substrate, releasing GMP and forming a phosphodiester bond (Fig. 1) [12, 13]. RtcB enzymes require two divalent metal ions to proceed with RNA ligation. The enzyme coordinates one ion before reacting with GTP. The second ion is required for proper coordination of GTP. No binding of GTP to RtcB is observed in the absence of metal ions [12].

Human RtcB was first identified in human HeLa cell extracts, which possess a 3′–5′ RNA ligase activity between 2′,3′-cyclic phosphate and 5′-OH termini [14]. This RNA ligase activity was found to be dependent on 5′-OH and 2′,3′-cyclic phosphate RNA-ends. Further investigations using a combination of chromatographic purification, ligase activity assays, mass spectrometry and phylogenetic analysis led to identification of RtcB as the enzyme responsible for this activity [3, 15]. Additional experiments confirmed that the 2′,3′-terminal phosphate was retained in the re-ligated RNA product, indicating that RtcB’s RNA ligation mechanism is conserved from prokaryotes to human cells.

Although both 2′–3′ cyclic phosphate and 3′-phosphate ends can undergo ligation by RtcB, it seems that the former is the *bona fide* substrate as suggested by RtcB’s reduced efficiency with 3′-phosphate ends and the observation of an RNA 3′-terminal phosphate cyclase activity in HeLa cell extracts that converts the 3′-phosphate into 2′,3′-cyclic phosphate [14, 16]. In contrast, hydrolysis of 2′–3′ cyclic phosphate to 2′-phosphate by 2′–3′ cyclic nucleotide phosphodiesterase (CNP) decreases 3′–5′ RNA ligase activity by reducing levels of the substrate [17]. The presence of enzymes such as the RNA 3′-terminal phosphate cyclase A (RtcA) that convert a 2′-phosphate at the end of an RNA into a 2′–3′ cyclic phosphate end counteracts CNP activity and facilitates ligation of such RNAs by RtcB [17]. Conversely, the 2′–3′ cyclic phosphatase activity of human ANGEL2, and to a lesser extent ANGEL1, can convert 2′–3′ cyclic phosphate at the terminus of an RNA into 2′- or 3′-hydroxylated groups, thus reducing 3′–5′ ligase activity [18].

### Crystal structure

Comparative genomic and structural studies show that RtcB proteins are evolutionarily conserved. For example, human RtcB shares 51% amino acid identity with RtcB from the archaeon *Pyrococcus horikoshii* and 73% with the nematode *Caenorhabditis elegans* [19, 20]. Moreover, crystal structures of RtcB from different archaeal, bacterial and eukaryotic species show a unique but conserved protein fold [19, 21].

**Fig. 2:**
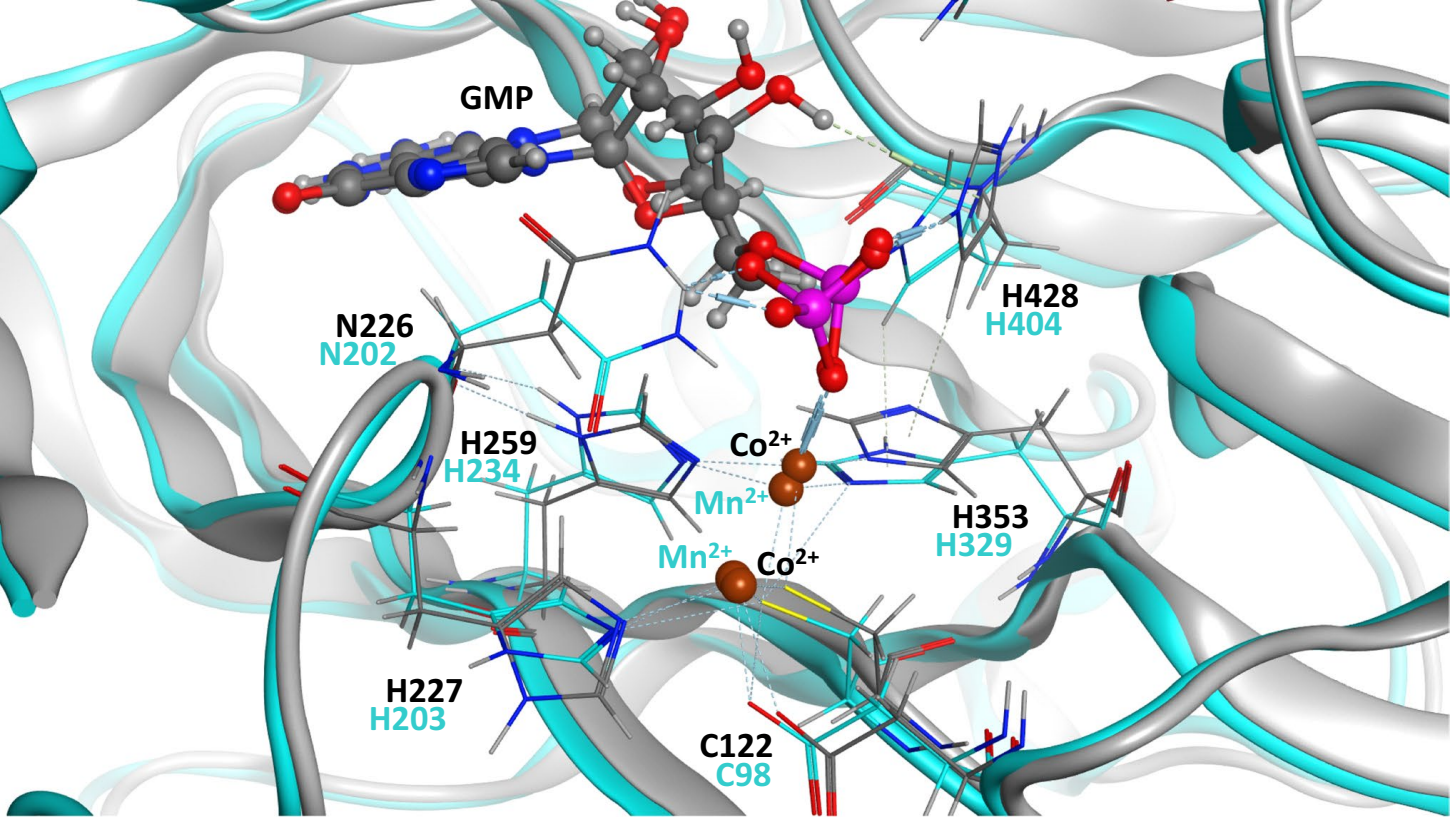
RtcB catalytic site crystal structure. Superposition of *H. sapiens* (grey) and *P. horikoshii* (cyan) RtcB catalytic site crystal structures. The high homology between the two catalytic sites demonstrates that both orthologues share the same ligation mechanism with a covalently bound GMP but with different divalent cation specificity. Here, *H. sapiens* and *P. horikoshii* RtcB crystal structures are represented in complex with Co^2+^ and Mn^2+^ ions, respectively. Key amino acids for RtcB RNA ligation activity are depicted in black for *H. sapiens* and cyan for *P. Horikoshii*

Several crystal structures of RtcB proteins from the archaeon *Pyrococcus horikoshii*, *Pyrobaculum aerophilum and* the bacteria *Escherichia coli* have been solved. These help shed light on the mechanism of action of RtcB, identifying key amino acids in the catalytic site responsible for coordination of the metal ions to achieve RNA ligation (Fig. 2). Indeed, sequence alignment between *Escherichia coli*, *Methanocaldococcus jannaschii*, *Caenorhabditis elegans* and *Homo sapiens* revealed six conserved histidine residues which could coordinate a metal ion involved in RtcB function [22]. Further analysis of the structure of RtcB from *P. horikoshii* revealed a hydrophilic pocket where Cys98, His203, His234 and His404 are in close proximity. Comparison of this structure with proteins in the Protein Data Bank showed similarities with several zinc metalloenzymes also presenting adjacently positioned cysteine and histidines, initially suggesting a requirement for Zn^2+^ ions in RtcB function [21]. It is relevant to note that alanine substitution mutations of the three corresponding residues in RtcB of *P. aerophilum,* i.e. Cys100, His205 or His236, and of the conserved Cys78 and Cys122 residue in *E. coli* and human RtcB, respectively, produced ligase-dead proteins [2, 3, 23].

The involvement of metal ions in RtcB RNA ligation has been a subject of particular attention. In 2012, two crystal structures of *P. horikoshii* RtcB, one in complex with Mn^2+^ ions and one in complex with Mn^2+^ and GMP, showed that binding of the nucleotide to His404 induces a conformational change in the structure of RtcB [24]. These models suggested that one of the Mn^2+^ ions used for overall RNA ligation can be substituted with Zn^2+^, while the other Mn^2+^ ion is required for guanylylation of RtcB and cannot be substituted with Zn^2+^, thus introducing to the field a requirement for two different divalent metal ions for RtcB guanylylation [24]. The following year, another structural study in *P. horikoshii* revealed how the two Mn^2+^ ions coordinate GTP, with one Mn^2+^ linked to Cys98, His234 and His329, coordinating the α-phosphate of GTP and the second Mn^2+^ linked to Asp95, Cys98 and His203 contacting the γ-phosphate of GTP [13]. Additional experiments revealed that RtcB orthologues have different specificity for metal ions for RtcB guanylylation. For example, *E. coli* RtcB has the most stringent requirements, being active in the presence of Mn^2+^ ions only [23]. In contrast, the crystal structure of *P. horikoshii* revealed that RtcB guanylylation, while likewise most effective with Mn^2+^, also accepts Co^2+^ and Ni^2+^ ions [25]. The reasons for this discrepancy between RtcB orthologues are not fully understood as the amino acids serving as ligands for these metal ions are conserved. It has been suggested that there may be differences in the metal coordination geometry between the metallic ions [25]. Jacewicz et al. has recently reviewed the current knowledge about RtcB metal ion usage, both for guanylylation and phosphodiester synthesis steps [25].

Recently, the crystal structure of human RtcB was solved [26]. This structure, which was determined at a resolution of 2.3 Å, reveals RtcB in complex with GMP and two divalent Co^2+^ ions. Human RtcB was shown to catalyse the ligation reaction with high efficiency not only in the presence of Co^2+^ and Mn^2+^ ions, but also in the presence of Mg^2+^ and Zn^2+^, albeit to a lesser extent [26]. However, as suggested above, the coordination of the Mn^2+^ ions in the catalytic site of RtcB may be different from that of Co^2+^ ions since RtcB from *E. coli* exhibited strong ligase activity in the presence of Mn^2+^ ions, which was abrogated by Co^2+^ ions [20]. The capacity of human RtcB to catalyse the ligation reaction with either Mn^2+^ or Co^2+^ ions, despite the change in metal coordination geometry between *P. horikoshii* Mn^2+^-RtcB-GMP and human Co^2+^-RtcB-GMP structures, demonstrates a superior structural plasticity of the human RtcB-GMP-binding site, and suggests that the metal ion coordination geometry in human RtcB might be more dynamic and susceptible to change during the catalytic cycle to support every step of the ligation mechanism [26].

In summary, two major questions remain unanswered regarding the activity of RtcB orthologues. First, the modalities of metal ion binding and coordination in the catalytic site, which determines their impact on RtcB guanylylation and phosphodiester synthesis, are not fully understood. Results from human RtcB, suggesting that the coordination of these ions might be dynamic during the RtcB catalytic cycle, and the metal mixing experiments using combinations of Mn^2+^ and other acceptable metal ions under physiological conditions indicate a more complex system than previously suggested by crystal structures alone. Second, the pose of RNA fragments in the catalytic site and their interactions with GMP, metal ions and the residues of RtcB remain to be described. While previous publications pinpointed specific amino acid residues involved in 3′ or 5′ RNA fragment binding in *E. coli* and *P. horikoshii*, more needs to be done to fully describe RtcB ligation strategy, especially in the human ligase [24, 27, 28].

## Regulation of RtcB

The ligase activity of RtcB can be regulated in several ways including subcellular localisation, interaction with other proteins, post-translational modifications and oxidation. These will be discussed below.

### Regulation of localisation

**Fig. 3:**
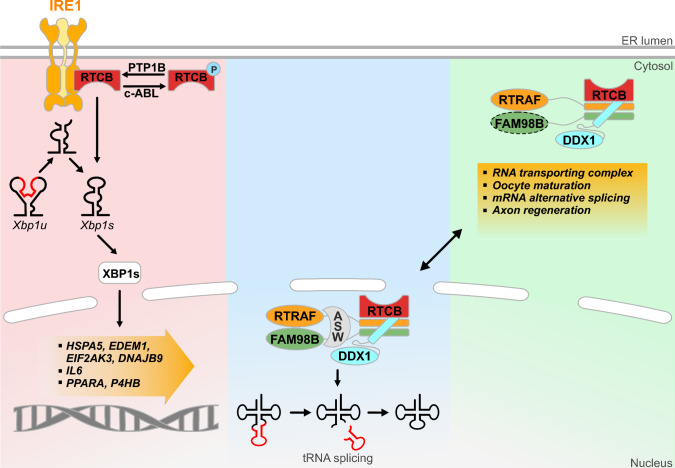
Overview of pathways linked to RtcB activity. RtcB can be found in the cytoplasm and the nucleus, enabling it to achieve its functions. [Left] In the cytoplasm, induction of the unfolded protein response in the ER leads to activation of IRE1, which cuts XBP1 mRNA at two stem-loop structures, removing a 26-nucleotide intron. The 5′ and 3′ ends of the cut fragments are ligated with RtcB generating a new mRNA termed spliced XBP1 (XBP1s). Then, the XBP1s transcription factor induces expression of genes involved in protein folding, immunity and lipids regulation. [Middle] In the nucleus, intron-containing pre-tRNAs have to undergo non-conventional splicing events, removing the intron and generating two exon halves. Then, RtcB tRNA ligase activity re-ligates the exon halves, generating a mature tRNA for protein translation. [Right] RtcB has XBP1- and tRNA-independent functions, which remain to be fully elucidated as the RtcB targets linked to these functions and their cellular localisation remain to be identified. These functions could require the presence of some members of the tRNA ligase complex, of which RtcB, RTRAF and DDX1 have been detected in the cytoplasm with and without FAM98B (dotted line)

RtcB is found in both the nucleus and in the cytosol, corresponding to its roles in tRNA ligation and in maturation of XBP1s mRNA, respectively [29]. In the nucleus, RtcB is found as part of a complex called the tRNA ligase complex [26] that includes RtcB and four associated proteins: DDX1 (DEAD-box helicase 1), RTRAF (RNA transcription, translation and transport Factor, also known as hCLE, C14orf166 or CGI99), FAM98B (family with sequence similarity 98 member B) and Ashwin [3]. In the cytosol, human RtcB can be found associated with the ER and has been confirmed to interact physically with IRE1, although this interaction was not confirmed to be direct or as a part of a complex (Fig. 3) [5]. Thus, it may be part of the UPRosome, a collection of IRE1-interacting proteins that modify IRE1’s activity [30]. It is not known if any of the other components of the tRNA ligase complex are also part of the IRE1 interactome.

The core tRNA ligase complex consisting of RtcB, RTRAF, DDX1, FAM98B shuttles between nucleus and cytosol. The mechanism regulating RtcB movement between the nucleus and the cytosol remains elusive. RTRAF has been postulated as the shuttling factor due to various observations. It is found to be associated with myosin light chain kinase II, which modulates myosin II activity and is involved in the active transport to the cytoplasm. In addition, fluorescence microscopy shows that RTRAF moves between these two compartments and an association between RTRAF, RtcB, DDX1 and FAM98B has been confirmed in both compartments [31]. However, these observations do not exclude the possibility that other protein(s) may also fulfil this role as no functional assays definitely confirmed the role of RTRAF as the driver of shuttling of the complex. Another potential candidate is Ashwin, which possesses two nuclear localisation signals and a high percentage of basic amino acids typical of nuclear proteins [32]. Notably, Ashwin has not been found in cytosolic RtcB complexes. It is possible that the nuclear localisation is controlled by Ashwin, while RTRAF controls cytoplasmic translocation. Interestingly, inhibition of transcription with actinomycin D reduces translocation of RtcB, DDX1 and RTRAF to the nucleus and leads to their accumulation in the cytosol, though the mechanism how transcription may regulate the localisation of these proteins is not clear [31]. Further investigations may reveal whether localisation of RtcB is regulated by post-translational modifications, e.g. phosphorylation, or cellular signalling, e.g. ER stress activation of the unfolded protein response.

### Post-translational modification

Recent studies show that RtcB can be regulated by phosphorylation in response to ER stress. Indeed, RtcB’s interaction with IRE1 seems to be under the control of post-translational modifications. In the cytoplasm, c-Abl can phosphorylate RtcB on tyrosine residues 306, 316 and 475 and PTP1B can dephosphorylate these residues (Fig. 3) [33]. Interestingly, tyrosine phosphorylation of RtcB increases upon treatment with tunicamycin, a widely used ER stressor; however, it is not known whether this is dependent on c-Abl. Structural modelling suggests that phosphorylation of Tyr475 abrogates binding of GTP to the catalytic site of RtcB and that phosphorylation of Tyr306 reduces the interaction between RtcB and IRE1. Concomitantly, it was shown that phosphorylation of Tyr306 reduces the levels of XBP1s and that cells expressing Y306F mutant RtcB have increased XBP1s production. Importantly, no change in tRNA ligation was observed in these mutant cells, showing that RtcB can be regulated post-translationally in a manner that affects its function in the IRE1 signalling pathway, but not its tRNA ligation activity [33].

### Regulation by Archease

The activity of RtcB is also regulated through interaction with Archease. Not all organisms express Archease, an observation which has provided much information on its role in regulating RtcB activity. In organisms that lack Archease, such as *Thermobifida fusca*, RtcB is a single-turnover enzyme, and cannot be improved catalytically by addition of Archease from another species [34]. In organisms such as humans where Archease is present, it converts RtcB into a multiple-turnover enzyme. In fact, human RtcB, which normally functions with Archease, cannot effectively accomplish re-ligation of *XBP1* in yeast cells lacking RtcB and requires co-expression of Archease to show full enzymatic activity [35]. By contrast, RtcB from *E. coli*, which does not require Archease, could effectively execute re-ligation reactions in some yeast-based systems [35]. The mechanism underlying this differential ability of Archease to affect RtcB activity between orthologues is currently unknown. A structural comparison of RtcB orthologues between species that do or do not express Archease could provide valuable insight into RtcB’s single/multiple-turnover properties and how Archease can impacts this aspect of RtcB activity.

In addition, Archease can alter the specificity of RtcB for nucleotides in the ligation reaction, such that in the presence of Archease, RtcB can use dGTP, ATP and ITP in addition to GTP. ATP in particular, was shown to stimulate RtcB guanylylation in presence of Archease, an effect that was dependent on the ability of DDX1 to bind and hydrolyse ATP [36]. In agreement with these observations, the multiple-turnover ability of RtcB is lost in the absence of DDX1 and RTRAF due to the concomitant depletion of DDX1 during RTRAF knockdown, suggesting that Archease works in collaboration with DDX1 to support RtcB activity. Moreover, Archease can rescue the catalytic activity of RtcB mutants in the GTP-binding pocket, suggesting Archease may have a nucleotide-binding pocket [37]. However, Archease could not be co-immunoprecipitated with RtcB or the tRNA ligase complex without the use of a crosslinking reagent [26, 34, 36], indicating a transient interaction, which is supported by the observation that Archease from *P. horikoshii* can enhance the catalytic activity of *P. horikoshii* RtcB in a sub-stoichiometric ratio [37].

Importantly, Archease is localised to both the cytosol and the nucleus, albeit at lower levels in the nucleus, and is functionally important for both XBP1 and tRNA ligation in human HeLa cells [4]. Its depletion by shRNA impairs RtcB-induced XBP1 ligation and, similarly, siRNA inhibition of Archease reduces RtcB-induced tRNA ligation [4, 36].

### Regulation by oxidative stress

There is some evidence that RtcB activity is sensitive to inactivation by oxidative stress. Murine cells with oxidative stress exhibit an accumulation of tRNA exon halves [38]. Later, it was observed that the essential cysteine residue that coordinates metal ions in the active site is oxidised in the crystal structure of RtcB aerobically purified from archaeal *P. horikoshii* [28]. More recently, PYROXD1, an oxidoreductase that co-evolved with RtcB, has been shown to protect RtcB from oxidative stress-induced inactivation by preventing oxidation of the conserved Cys122 in the catalytic site of human RtcB [39]. PYROXD1 oxidises NAD(P)H to NAD(P)^+^, which can suppress Cu-mediated inactivation of RtcB. However, the reaction also involves reduction of O_2_ to H_2_O_2_, thus generating undesirable H_2_O_2_ in the vicinity, which can inactivate RtcB. Thus, high levels of oxidative stress that deplete NAD(P)H can override this protective mechanism leading to inactivation of RtcB [39]. It is suggested that PYROXD1 likely operates within a narrow range to avoid accumulation of H_2_O_2_ that could inactivate RtcB. Since it is so tightly controlled, a regulatory function for the redox sensitivity of RtcB has been suggested, but not yet shown. For example, it could play a role as a molecular switch during oxidative stress coupled to ER stress.

## Key substrates of RtcB RNA ligase activity

### RtcB is the key component of the tRNA ligase complex

The first identified function for RtcB was in ligation of select tRNAs that require removal of an intron for maturation. In fact, in humans, 28 of 416 tRNAs contain introns, including all 13 tRNAs that transfer tyrosine to ribosomes [40]. Thus, RtcB is crucial for general translation.

In metazoans, RtcB accomplishes tRNA ligation as part of a multi-subunit ligase complex called the tRNA ligase complex. The human tRNA ligase complex was identified through activity-guided purification of tRNA ligase activity from HeLa cell extracts, revealing a complex containing RtcB and DDX1, RTRAF, FAM98B and Ashwin [3]. Further in vitro analysis, depleting each of the proteins of the complex, confirmed RtcB as the essential subunit that possesses enzymatic activity. The other members of the complex are diverse and not restricted to the tRNA ligase complex. DDX1 is an ATP-dependent RNA helicase involved in DNA double-strand break repair and micro-RNA maturation [41, 42]. Biochemical assays investigating RtcB guanylylation revealed that although ATP is not used by RtcB as a nucleotidyl donor, ATP is required for full guanylylation. In fact, mutation of DDX1 at a conserved residue (K52N) affects RtcB guanylylation [36]. RTRAF is a multimeric protein that is homologous to proteins of the FACT transcription regulatory complex [31, 43]. Further investigations indicated a role in transcription through its co-localisation with RNA polymerase II and a 50% inhibition of global RNA transcription upon RTRAF silencing in MDCK and HEK293T cells respectively [43]. However, its function in the tRNA ligase complex is unknown but may be related to localisation, as discussed earlier. Almost nothing is known about FAM98B other than the finding that it is in the tRNA ligase complex. Information about Ashwin is similarly scarce. However, as suggested earlier it may contribute to nuclear localisation of the complex. Its only known function is in Xenopus embryo development, during which it regulates cell survival and anteroposterior patterning [32]. The functions of FAM98B and Ashwin in the tRNA ligase complex remain to be elucidated.

Interestingly, RtcB and some of the other subunits of tRNA ligase complex have been reported to also be part of other protein complexes, suggesting a dynamic relationship between the components. For example, RtcB, DDX1 and RTRAF are associated with kinesin 5 as part of an RNA-transporting complex in mouse neurons [44]. Additionally, RtcB, RTRAF, DDX1 and FAM98B were found to be associated with mRNA caps, implicating these components of the complex in the transport and translation of RNA granules in HEK293T [31, 45]. Furthermore, it has been shown recently that RTRAF and FAM98B can form heterodimers, independent of the presence of other subunits of the complex, although the function of these heterodimers is as yet unknown [26].

### RtcB in XBP1s maturation

Identification of RtcB as the ligase responsible for *XBP1* mRNA maturation was demonstrated subsequent to reports of its role in tRNA ligation [4, 5, 20]. In response to accumulation of unfolded proteins in the ER, IRE1 is activated and its RNAse activity cleaves two stem-loop structures in *XBP1* mRNA, resulting in the removal of a 26-nucleotide intron. The 5′ and 3′ mRNA fragments produced are re-ligated by RtcB [4, 5, 20]. This splicing event provokes a frameshift enabling the translation of XBP1s, which is a potent transcription factor. The maturation of XBP1 mRNA into its spliced active form is central for IRE1 signalling in protein and lipid homeostasis (Fig. 3) [8–11].

It is not clear which, if any, of the components of the tRNA ligase complex are required for XBP1 splicing apart from RtcB itself. While a complex containing RtcB, RTRAF, DDX1 and FAM98B can be found in both the nucleus and the cytosol [31], this does not necessarily mean that all of these components are required for ligation of *XBP1* mRNA fragments. In fact, XBP1 splicing, including cleavage and rejoining of the cleaved fragments can be reconstituted in vitro with just recombinant IRE1 (which performs the double cleavage of *XBP1* mRNA at two stem loops) and RtcB, although only about 50% of the cleaved XBP1 exons were rejoined [5]. It may be that other components of the tRNA ligase complex found in the cytosol are involved in trafficking RtcB between the nucleus and cytosol or in increasing the efficiency of the reaction.

## RtcB functions: from bacteria to humans

Much of what we understand about RtcB function and regulation in human cells is inferred from observations in other organisms including prokaryotes. RtcB has a major impact on translation in cells due to its roles in tRNA and XBP1s maturation. It can directly affect translation at the ribosomal level by regulating the levels of tRNAs that require splicing. It also exerts an indirect effect on protein expression through production of XBP1s, which maintains ER homeostasis due to the expression of its target genes. Moreover, RtcB may also target other unidentified RNA substrates, linking RtcB to other functions of which we are currently unaware. Here, we summarise what is known about the consequences of RtcB activity in various biological systems, from bacteria to human cells and finish by examining the potential of RtcB as a therapeutic target in human cancer.

### RtcB and genotoxic and ribosomal stress in bacteria

The functional importance of RtcB during genotoxic and ribosomal stresses was uncovered through studies on the bacterial operon encoding the orthologs RtcA and RtcB [46]. Ribotoxin, abiotic stressors and genetic lesions impairing the translation apparatus or causing oxidative damage were all shown to activate expression of the Rtc operon in *E. coli* [47]. Moreover, Rtc deletion produced transcriptomic changes that were linked to non-protein coding RNA (rRNA, tRNA, sRNA), ribosomal proteins, amino acid biosynthesis and transport. Impaired ribosome profiles were due to degradation of rRNA in RtcB mutants, a phenotype which could be rescued by ectopic overexpression of RtcB [47]. Further analysis of RtcB functions in *E. coli* suggests involvement in re-ligation of damaged rRNA. RtcB deletion leads to diminished recovery from ribosomal damage after exposure to antibiotic-induced stress conditions [48]. RNA-seq results confirmed an increase in cleaved rRNA in RtcB KO strains, again indicating involvement of RtcB in repair of cleaved rRNAs. In fact, RtcB was shown to be required for the recovery of *E. coli* upon stress release, due to its ability to re-ligate rRNA in ribosomes modified by endoribonuclease MazF as part of the stress response [49]. In addition, RtcB defects during oxidative stress revealed 55 degraded transcripts, among which tRNA-coding genes were overrepresented [50]. Further analysis using an adapter sequence in RtcB ligation reactions on cleaved RNA extract, identified 34 potential RtcB targets, among which ssrA, a transfer-messenger RNA (tmRNA), was the most frequently found. ssrA is involved in trans-translation, a process preserving ribosome recycling in response to translation defects such as broken mRNA, absence of appropriate tRNA or mRNA lacking a stop codon, further confirming the role of RtcB in the preservation of ribosomes [50, 51].

Other studies support a role for RtcB in ribosomal stress and DNA repair beyond its classical role in RNA ligation. It has been suggested that the survival advantage conferred by RtcB under conditions of DNA damage in *Salmonella enterica Serovar Typhimurium* may be due to RtcB DNA-capping activity [52]. In *E. coli*, RtcB can produce a DNA_3′_pp_5′_G at DNA broken ends and this RtcB-induced DNA cap could then protect the 3′end of *E. coli* DNA from exonucleases [53]. These observations are supported by analysis of *Myxococcus xanthus* RtcB paralogues, which demonstrate variable activities and/or efficiency levels for RNA/DNA ligation and capping [54]. Further studies would be required to assess whether similar roles for RtcB exist in human or other eukaryotic cells.

### RtcB in metazoan physiology

Genetic manipulation of RtcB (e.g. downregulation, inactivation, KO) in animal models represents an opportunity to decipher the role of RtcB activity in multicellular organisms. There are only a few studies in this area but they have revealed roles for RtcB in physiology, neurology and immunology.

In *C. elegans*, RtcB KO causes growth deficits and shorter life span, which can be rescued by expression of pre-spliced tRNA [20, 55]. Curiously, RtcB KO in *C. elegans* also causes defects in vulval development and sterility due to failure in oocyte maturation, which are independent of both XBP1 and tRNA maturation (Fig. 3) [20]. A similar phenotype was obtained in mice in which conditional RtcB knockout in oocytes resulted in female infertility [56]. Causation for oocyte development failure upon RtcB defect was attributed to the accumulation of intron-retaining mRNAs coding for epigenetic factors involved in DNA methylation and DNA damage repair proteins, leading to their decreased expression and to further transcriptomic changes impacting key events in oocyte maturation. Additionally, zygotes obtained from the mating of RtcB KO females and WT males resulted in embryonic development defects. Furthermore, YY1, a murine transcription factor shown to be essential for murine reproduction has been shown to be able to induce RtcB expression in the mouse uterus, linking RtcB activity to embryo implantation, uterine receptivity and decidualisation [57]. As the IRE1–XBP1 pathway was reported to be involved in mouse decidualization, this has been suggested to be the downstream pathway linking RtcB to these reproductive functions, although this hypothesis remains to be confirmed [58]. Finally, RtcB KD by RNAi in B6D2F1 mice induces placental dysfunction resulting in foetal death, illustrating the impact of RtcB activity from gamete cells to foetal development [59]. However, the mechanisms at play in these defects remain to be assessed. These examples of the consequences of RtcB depletion on physiology suggest that RtcB functions are not linked solely to tRNA and XBP1 splicing. As an RNA ligase, it is plausible that other RNA substrates could be targeted and re-ligated by RtcB, involving RtcB in mRNA maturation as suggested by increased intron retention in mRNA upon RtcB depletion.

Several studies demonstrate a role for RtcB in neuronal systems and neurodegeneration. In a *C. elegans* model of Parkinson’s disease, an RNAi screen was the first to link RtcB (F16A11.2) to a neurodegenerative disease [60]. Downregulation of RtcB led to enhanced age-associated aggregation of α-synuclein, hinting at RtcB as a potential neuroprotective factor. Later, it was reported that neurodegeneration in *C. elegans* due to α-synuclein overexpression and or treatment with the Parkinson mimetic 6-hydroxydopamine could be rescued with co-expression of RtcB [61]. RtcB-mediated neuroprotection was dependent on its ligase activity and the downstream expression of XBP1s. In contrast, RtcB was shown to inhibit axon regeneration after single-neuron laser axotomy in *C. elegans* [55]. This effect was dependent on RtcB ligase activity but was independent of its activity as a tRNA and XBP1 ligase. This is in agreement with previous reports showing positive effect of XBP1 on axon regeneration, suggesting other substrates [62]. Thus, RtcB seems to govern two downstream pathways, having a positive effect on neurodegeneration and axon regeneration through XBP1 on the one hand, and a negative effect on axon regeneration on the other, through an as yet unidentified substrate. While the mechanism underlying this effect on axon regeneration was not identified, the authors observed that the cellular localisation of RtcB shifted from the perinuclear compartment of healthy neurons to the tip of the injured axons [55]. Thus, the authors proposed that RNA ligation might inhibit regeneration at the site of injury, and its localisation at the tip of the axon could be due to its association with neuronal RNA trafficking granules [44]. These observations regarding the inhibitory effect of RtcB on axonal regeneration were complemented by the description of a positive impact of RtcA defects on axon regeneration in *Drosophila* and cultured mouse neurons [63]. In these models, RtcA was shown to act as an inhibitor of axon regeneration while Archease and the XBP1 pathway were confirmed to increase neuron regenerative potential. Together, these observations suggest that despite a beneficial effect of the RtcB–Archease–XBP1 pathway, RtcA and RtcB share a common activity, dependent on RtcB ligation activity but independent of tRNA and XBP1 ligation, which is detrimental to axon regeneration. The mechanism or specific target underlying this phenotype remains to be described.

In the immune system, XBP1s is well characterised as essential for differentiation of B lymphocytes into plasmocytes and subsequent antibody production [64, 65]. Accordingly, RtcB depletion by conditional KO has been shown to reduce antibody secretion in mouse B cells [4]. Further examination of this phenotype showed that RtcB KO B lymphocytes could differentiate into plasmocytes with a similar number of IgM-secreting cells compared to WT and heterozygous controls. However, ELISPOT analysis showed that RtcB KO cells had a reduced ability to secrete immunoglobulins. This defect was associated with a disorganised ER morphology similar to that previously described in an XBP1 KO mouse B cell model [66]. It was confirmed to be independent of RtcB tRNA ligase activity, as the RtcB KO plasmocytes did not display global changes in protein synthesis rates while the Igμ heavy chain expression was distinctly reduced. Altogether, these results demonstrate the expected functional overlap between RtcB and XBP1, at least in B cell biology. To our knowledge, no other publications specifically tested RtcB functions in the immune system. Nonetheless, it is expected that many of the already described defects in immune functions due to loss of XBP1 [67–69] would be reproduced by RtcB defects.

### RtcB and human cancer

Several lines of evidence support a potential role for RtcB in human cancer, and thus it may have value as a therapeutic target. RtcB has been shown to mediate resistance to antioestrogen treatment in MCF7 breast cancer cells [70]. Mass spectrometry analysis revealed an enrichment of RtcB in protein aggregates induced by antioestrogen treatment. In this model, resistant and sensitive cells could be distinguished by their variable tendency to form aggresomes, with resistant cells capable of preventing their accumulation through maintenance of high XBP1s activity. Moreover, siRNA targeting RtcB as well as IRE1 inhibitors reversed the resistance of MCF7 cells to tamoxifen. Further analysis from patient samples confirmed that protein aggregation could predict tumour sensitivity to therapy, as RtcB showed lower co-localisation with aggresomes in the metastatic versus primary tumours prior to antioestrogen treatment, confirming a role for RtcB in treatment resistance.

Two independent studies identified RtcB as a target of miR-34a in cancer. A miRNA microarray analysis found miR-34a to be the most downregulated miRNA in breast cancer cell lines compared to healthy mammary epithelial cell lines [71]; and an upregulated target of genistein in head and neck cancer tumour-initiating cells (HNC-TIC) [72]. In the former, overexpression of miR-34a was able to inhibit tumour-initiating properties of breast cancer stem cells and to sensitise MDA-MB-231 cells to docetaxel by targeting RtcB expression [71]. Furthermore, higher RtcB expression was shown to correlate with shorter disease-free survival (DFS) and reduced overall survival (OS) in patients. Accordingly, patients with higher miR-34a expression had more favourable DFS and OS. In HNC-TIC, increased miR-34a expression through both direct transfection and genistein induction was able to reduce RtcB expression and the malignant phenotype (defined by increased self-renewability, invasion capacity and colony-forming property) [72]. Consistent with this, mouse xenografts confirmed the anti-tumour effect of targeting RtcB, as the tumour volume and weight were reduced in a dose-dependent manner. Similar to what was shown in breast cancer [71], a negative correlation was also found between miR-34a and RtcB expression in HNC tissues from The Cancer Genome Atlas (TCGA) database. Despite the concordant results between these two publications, the RtcB downstream pathways underlying these pro-tumorigenic effects remain to be identified.

Taken together, these studies show that the role of RtcB impacts various fields such as neurology, immunology and cancer. However, further studies are needed to elucidate many of these aspects.

## Conclusions

Since the discovery of RtcB as the enzyme responsible for RNA ligase activity in human cells, several groups tried to resolve the mechanisms governing its activity and its various functions. In this review, we summarised the findings obtained from three broad strategies to decipher RtcB functions: analysis of its crystal structure, biochemical analysis of its regulation and what is known about its functions in diverse organisms from bacteria to humans. However, there are still several key questions that remain unanswered.

While RtcB activity itself is now well known, thanks to the large number of studies describing its ligase activity, information about the specific functions of each of the tRNA ligase complex subunits is relatively patchy. Regarding DDX1, while its roles as a helicase and as a guanylylation factor of RtcB have been recognised, its importance for the proper positioning of RNA fragments in the catalytic site of RtcB is mostly speculative and moreover, its knockdown does not completely abrogate RNA ligation. Similarly, RTRAF knockdown causes a reduction of RNA ligation in HeLa cells, an effect that was attributed to a concomitant loss of DDX1 without excluding a potential functional role of this protein in the complex [36]. While some reports have described its ability to migrate between cytosol and nucleus, suggesting that RTRAF could be responsible for shuttling of the tRNA ligase complex between these compartments, this hypothesis remains to be confirmed, as well as the RTRAF shuttling mechanism itself [31]. In addition, thus far there are no reports on the role of FAM98B and Ashwin in cellular processes relevant to RtcB, as knockdown of FAM98B or Ashwin does not appear to affect maturation of tRNAs [36]. Finally, although not a *bona fide* subunit in the complex, investigation into Archease’s ability to increase RtcB RNA ligation activity by converting it into a multiple-reaction enzyme is a worthwhile pursuit, albeit technically difficult due to the transient nature of its interaction with the tRNA ligase complex.

Interestingly, although RtcB has been mostly studied in the context of tRNA maturation and *XBP1* mRNA splicing, some studies involving defective RtcB excluded an involvement of both tRNA and XBP1 ligation in the induced phenotype [20, 62]. Thus, it is possible that RtcB has other, as yet unidentified, RNA targets or functions in human cells. This suggestion is further supported by the description of two new roles for RtcB in bacteria, first as an rRNA ligase to accelerate bacteria recovery after stress and second, as a DNA-capping enzyme to protect DNA-free ends after genotoxic stress [49, 52, 53]. Indeed the RNA ligase capability of *E. Coli* RtcB has been used to identify cleaved mRNA fragments with 5′-OH ends in yeast, revealing multiple mRNA targets as well as *HAC1*, the yeast homologue of *XBP1*, demonstrating that such strategy could be used to detect these aforementioned unidentified RtcB targets [73].

Finally, observations of the role of RtcB in multicellular organisms indicate a role in neurobiology, immunology and cancer, three areas linked to dysregulation of ER homeostasis [74–76]. Although this observation is not surprising due to the direct involvement of RtcB ligation activity on XBP1, it highlights the potential of RtcB as a therapeutic target especially in cancer. Recent reports show that the IRE1–XBP1 pathway, either due to constitutive activity of IRE1 or to activation of IRE1 induced by chemotherapeutic drugs, favours the survival of cancer cells [77]. Thus, inhibitors of RtcB ligase activity could sensitise cancer cell to cell death by blocking IRE1–XBP1 pro-survival effects.

However, targeting RtcB ligase activity as a treatment for cancer may present significant challenges for several reasons. First, the binding of GMP to the catalytic core of RtcB might make the ligase activity of RtcB virtually undruggable. A similar challenge has been encountered in the long-standing difficulties in targeting RAS in cancer [78]. However, other RtcB sites may be targetable such as the RNA-binding sites. Second, targeting RtcB ligase activity will have an impact on not just the XBP1 pathway but also on tRNA maturation and other unidentified RtcB RNA substrates, potentially leading to unexpected side effects such as the production of tRNA-derived stress-induced RNAs (tiRNAs) [79] which have been demonstrated to prevent apoptosis and are considered as promising biomarkers for cancer [80]. Nonetheless, alternative strategies can be considered. Indeed, targeting RtcB dephosphorylation by PTP1B might allow for abrogation of the XBP1 pathway while preserving tRNA ligation [33]. Conversely, sequestrating RtcB in the nucleus by inhibiting its translocation to the cytoplasm may represent a good alternative to achieve selective inhibition of XBP1s production without affecting tRNA ligation. In addition, partial reduction of RtcB activity could also be of interest by targeting its association with Archease or with other proteins of the tRNA ligase complex. Regardless of these challenges, it is clear that RtcB may play hitherto unsuspected roles in cancer that are worthy of further investigation.

## Data Availability

Data sharing is not applicable to this article as no new data were created or analysed in this study.

## Abbreviations

ANGEL1 & 2: Angel Homolog 1 and 2
ATP: Adenosine triphosphate
*C. elegans*: *Caenorhabditis elegans*
CNP: Cyclic nucleotide phosphodiesterase
DDX1: DEAD-box helicase 1
DFS: Disease-free survival
dGTP: Deoxyguanosine triphosphate
*E. coli*: *Escherichia coli*
ELISPOT: Enzyme-linked immunospot
FACT: Facilitates chromatin transcription
FAM98B: Family with sequence similarity 98 member B
GMP: Guanosine monophosphate
GTP: Guanosine triphosphate
HEK293T: Human embryonic kidney 293 T
HNC-TIC: Head and neck cancer tumour-initiating cells
HSPC117: Hematopoietic stem/progenitor cell 117
IRE1α: Inositol-requiring enzyme 1 alpha
ITP: Inosine triphosphate
KD: Knockdown
MCF7: Michigan Cancer Foundation-7
MDCK: Madin–Darby canine kidney cells
miRNA: Micro-RNA
*M. jannaschii*: *Methanocaldococcus jannaschii*
mTOR: Mammalian target of rapamycin
NADPH: Nicotinamide adenine dinucleotide phosphate
OS: Overall survival
*P. aerophilum*: *Pyrobaculum aerophilum*
*P. horikoshii*: *Pyrococcus horikoshii*
PTP1B: Protein-tyrosine phosphatase 1B
PYROXD1: Pyridine nucleotide-disulphide oxidoreductase domain 1
RtcA: RNA 3′-terminal phosphate cyclase A
RtcB: RNA 2′,3′-cyclic phosphate and 5′-OH ligase
RNAi: RNA interference
rRNA: Ribosomal ribonucleic acid
RTRAF: RNA transcription, translation and transport factor
siRNA: Small interfering RNA
ssrA: Small stable RNA A
TCGA: The Cancer Genome Atlas
tiRNA: TRNA-derived stress-induced RNA
tmRNA: Transfer-messenger RNA
XBP1: X-box-binding protein 1
YY1: YY1 transcription factor

## References

[1] Yuan Y (2023). Chemoproteomic discovery of a human RNA ligase. Nat Commun.

[2] Englert M, Sheppard K, Aslanian A, Yates JR, Soll D (2011). Archaeal 3′-phosphate RNA splicing ligase characterization identifies the missing component in tRNA maturation. Proc Natl Acad Sci U S A.

[3] Popow J (2011). HSPC117 is the essential subunit of a human tRNA splicing ligase complex. Science.

[4] Jurkin J (2014). The mammalian tRNA ligase complex mediates splicing of XBP1 mRNA and controls antibody secretion in plasma cells. EMBO J.

[5] Lu Y, Liang FX, Wang X (2014). A synthetic biology approach identifies the mammalian UPR RNA ligase RtcB. Mol Cell.

[6] Yoshida H, Matsui T, Yamamoto A, Okada T, Mori K (2001). XBP1 mRNA is induced by ATF6 and spliced by IRE1 in response to ER stress to produce a highly active transcription factor. Cell.

[7] Lee K (2002). IRE1-mediated unconventional mRNA splicing and S2P-mediated ATF6 cleavage merge to regulate XBP1 in signaling the unfolded protein response. Genes Dev.

[8] Lee AH, Iwakoshi NN, Glimcher LH (2003). XBP-1 regulates a subset of endoplasmic reticulum resident chaperone genes in the unfolded protein response. Mol Cell Biol.

[9] Acosta-Alvear D (2007). XBP1 controls diverse cell type- and condition-specific transcriptional regulatory networks. Mol Cell.

[10] Fang P (2018). IRE1alpha-XBP1 signaling pathway regulates IL-6 expression and promotes progression of hepatocellular carcinoma. Oncol Lett.

[11] Moncan M, Mnich K, Blomme A, Almanza A, Samali A, Gorman AM (2021). Regulation of lipid metabolism by the unfolded protein response. J Cell Mol Med.

[12] Desai KK, Raines RT (2012). tRNA ligase catalyzes the GTP-dependent ligation of RNA with 3′-phosphate and 5′-hydroxyl termini. Biochemistry.

[13] Desai KK, Bingman CA, Phillips GN, Raines RT (2013). Structures of the noncanonical RNA ligase RtcB reveal the mechanism of histidine guanylylation. Biochemistry.

[14] Filipowicz W, Konarska M, Gross HJ, Shatkin AJ (1983). RNA 3′-terminal phosphate cyclase activity and RNA ligation in HeLa cell extract. Nucleic Acids Res.

[15] Galperin MY, Koonin EV (2004). ‘Conserved hypothetical’ proteins: prioritization of targets for experimental study. Nucleic Acids Res.

[16] Vicente O, Filipowicz W (1988). Purification of RNA 3′-terminal phosphate cyclase from HeLa cells. Covalent modification of the enzyme with different nucleotides. Eur J Biochem.

[17] Unlu I, Lu Y, Wang X (2018). The cyclic phosphodiesterase CNP and RNA cyclase RtcA fine-tune noncanonical XBP1 splicing during ER stress. J Biol Chem.

[18] Pinto PH (2020). ANGEL2 is a member of the CCR4 family of deadenylases with 2′,3′-cyclic phosphatase activity. Science.

[19] Nandy A, Saenz-Mendez P, Gorman AM, Samali A, Eriksson LA (2017). Homology model of the human tRNA splicing ligase RtcB. Proteins.

[20] Kosmaczewski SG (2014). The RtcB RNA ligase is an essential component of the metazoan unfolded protein response. EMBO Rep.

[21] Okada C, Maegawa Y, Yao M, Tanaka I (2006). Crystal structure of an RtcB homolog protein (PH1602-extein protein) from *Pyrococcus horikoshii* reveals a novel fold. Proteins.

[22] Genschik P, Drabikowski K, Filipowicz W (1998). Characterization of the *Escherichia coli* RNA 3′-terminal phosphate cyclase and its sigma54-regulated operon. J Biol Chem.

[23] Tanaka N, Chakravarty AK, Maughan B, Shuman S (2011). Novel mechanism of RNA repair by RtcB via sequential 2′,3′-cyclic phosphodiesterase and 3′-Phosphate/5′-hydroxyl ligation reactions. J Biol Chem.

[24] Englert M (2012). Structural and mechanistic insights into guanylylation of RNA-splicing ligase RtcB joining RNA between 3′-terminal phosphate and 5′-OH. Proc Natl Acad Sci U S A.

[25] Jacewicz A, Dantuluri S, Shuman S (2022). Structures of RNA ligase RtcB in complexes with divalent cations and GTP. RNA.

[26] Kroupova A (2021). Molecular architecture of the human tRNA ligase complex. Elife.

[27] Maughan WP, Shuman S (2016). Distinct contributions of enzymic functional groups to the 2′,3′-cyclic phosphodiesterase, 3′-phosphate guanylylation, and 3′-ppG/5′-OH ligation steps of the *Escherichia coli* RtcB nucleic acid splicing pathway. J Bacteriol.

[28] Banerjee A, Goldgur Y, Shuman S (2021). Structure of 3′-PO4/5′-OH RNA ligase RtcB in complex with a 5′-OH oligonucleotide. RNA.

[29] Lund E, Dahlberg JE (1998). Proofreading and aminoacylation of tRNAs before export from the nucleus. Science.

[30] Urra H, Pihan P, Hetz C (2020). The UPRosome—decoding novel biological outputs of IRE1alpha function. J Cell Sci.

[31] Perez-Gonzalez A, Pazo A, Navajas R, Ciordia S, Rodriguez-Frandsen A, Nieto A (2014). hCLE/C14orf166 associates with DDX1-HSPC117-FAM98B in a novel transcription-dependent shuttling RNA-transporting complex. PLoS ONE.

[32] Patil SS, Alexander TB, Uzman JA, Lou CH, Gohil H, Sater AK (2006). Novel gene ashwin functions in Xenopus cell survival and anteroposterior patterning. Dev Dyn.

[33] Papaioannou A (2022). Stress-induced tyrosine phosphorylation of RtcB modulates IRE1 activity and signaling outputs. Life Sci Alliance.

[34] Desai KK, Beltrame AL, Raines RT (2015). Coevolution of RtcB and Archease created a multiple-turnover RNA ligase. RNA.

[35] Poothong J, Tirasophon W, Kaufman RJ (2017). Biosci Rep.

[36] Popow J, Jurkin J, Schleiffer A, Martinez J (2014). Analysis of orthologous groups reveals archease and DDX1 as tRNA splicing factors. Nature.

[37] Desai KK, Cheng CL, Bingman CA, Phillips GN, Raines RT (2014). A tRNA splicing operon: Archease endows RtcB with dual GTP/ATP cofactor specificity and accelerates RNA ligation. Nucleic Acids Res.

[38] Hanada T (2013). CLP1 links tRNA metabolism to progressive motor-neuron loss. Nature.

[39] Asanovic I (2021). The oxidoreductase PYROXD1 uses NAD(P)(+) as an antioxidant to sustain tRNA ligase activity in pre-tRNA splicing and unfolded protein response. Mol Cell.

[40] Chan PP, Lowe TM (2009). GtRNAdb: a database of transfer RNA genes detected in genomic sequence. Nucleic Acids Res.

[41] Li L (2016). DEAD box 1 facilitates removal of RNA and homologous recombination at DNA double-strand breaks. Mol Cell Biol.

[42] Han C (2014). The RNA-binding protein DDX1 promotes primary microRNA maturation and inhibits ovarian tumor progression. Cell Rep.

[43] Perez-Gonzalez A, Rodriguez A, Huarte M, Salanueva IJ, Nieto A (2006). hCLE/CGI-99, a human protein that interacts with the influenza virus polymerase, is a mRNA transcription modulator. J Mol Biol.

[44] Kanai Y, Dohmae N, Hirokawa N (2004). Kinesin transports RNA: isolation and characterization of an RNA-transporting granule. Neuron.

[45] Pazo A, Perez-Gonzalez A, Oliveros JC, Huarte M, Chavez JP, Nieto A (2019). hCLE/RTRAF-HSPC117-DDX1-FAM98B: a new cap-binding complex that activates mRNA translation. Front Physiol.

[46] Das U, Shuman S (2013). 2′-Phosphate cyclase activity of RtcA: a potential rationale for the operon organization of RtcA with an RNA repair ligase RtcB in *Escherichia coli* and other bacterial taxa. RNA.

[47] Engl C, Schaefer J, Kotta-Loizou I, Buck M (2016). Cellular and molecular phenotypes depending upon the RNA repair system RtcAB of *Escherichia coli*. Nucleic Acids Res.

[48] Manwar MR (2020). The bacterial RNA ligase RtcB accelerates the repair process of fragmented rRNA upon releasing the antibiotic stress. Sci China Life Sci.

[49] Temmel H (2017). The RNA ligase RtcB reverses MazF-induced ribosome heterogeneity in *Escherichia coli*. Nucleic Acids Res.

[50] Kotta-Loizou I (2022). The RNA repair proteins RtcAB regulate transcription activator RtcR via its CRISPR-associated Rossmann fold domain. iScience.

[51] Himeno H, Kurita D, Muto A (2014). tmRNA-mediated trans-translation as the major ribosome rescue system in a bacterial cell. Front Genet.

[52] Kurasz JE (2018). Genotoxic, metabolic, and oxidative stresses regulate the RNA repair operon of *Salmonella enterica* serovar typhimurium. J Bacteriol.

[53] Das U, Chauleau M, Ordonez H, Shuman S (2014). Impact of DNA3′pp5′G capping on repair reactions at DNA 3′ ends. Proc Natl Acad Sci U S A.

[54] Maughan WP, Shuman S (2015). Characterization of 3′-phosphate RNA ligase paralogs RtcB1, RtcB2, and RtcB3 from *Myxococcus xanthus* highlights DNA and RNA 5′-phosphate capping activity of RtcB3. J Bacteriol.

[55] Kosmaczewski SG (2015). RNA ligation in neurons by RtcB inhibits axon regeneration. Proc Natl Acad Sci U S A.

[56] Zhang H (2022). The RNA ligase RNA terminal phosphate cyclase B regulates mRNA alternative splicing and is required for mouse oocyte development and maintenance. Development.

[57] Li R (2021). YY1 and RTCB in mouse uterine decidualization and embryo implantation. Reproduction.

[58] Gu XW (2016). Endoplasmic reticulum stress in mouse decidua during early pregnancy. Mol Cell Endocrinol.

[59] Wang Y (2010). HSPC117 deficiency in cloned embryos causes placental abnormality and fetal death. Biochem Biophys Res Commun.

[60] Hamamichi S, Rivas RN, Knight AL, Cao S, Caldwell KA, Caldwell GA (2008). Hypothesis-based RNAi screening identifies neuroprotective genes in a Parkinson’s disease model. Proc Natl Acad Sci U S A.

[61] Ray A, Zhang S, Rentas C, Caldwell KA, Caldwell GA (2014). RTCB-1 mediates neuroprotection via XBP-1 mRNA splicing in the unfolded protein response pathway. J Neurosci.

[62] Nix P, Hammarlund M, Hauth L, Lachnit M, Jorgensen EM, Bastiani M (2014). Axon regeneration genes identified by RNAi screening in *C. elegans*. J Neurosci.

[63] Song Y (2015). Regulation of axon regeneration by the RNA repair and splicing pathway. Nat Neurosci.

[64] Reimold AM (2001). Plasma cell differentiation requires the transcription factor XBP-1. Nature.

[65] Shaffer AL (2004). XBP1, downstream of Blimp-1, expands the secretory apparatus and other organelles, and increases protein synthesis in plasma cell differentiation. Immunity.

[66] Taubenheim N, Tarlinton DM, Crawford S, Corcoran LM, Hodgkin PD, Nutt SL (2012). High rate of antibody secretion is not integral to plasma cell differentiation as revealed by XBP-1 deficiency. J Immunol.

[67] Bettigole SE, Glimcher LH (2015). Endoplasmic reticulum stress in immunity. Annu Rev Immunol.

[68] Grootjans J, Kaser A, Kaufman RJ, Blumberg RS (2016). The unfolded protein response in immunity and inflammation. Nat Rev Immunol.

[69] Navid F, Colbert RA (2017). Causes and consequences of endoplasmic reticulum stress in rheumatic disease. Nat Rev Rheumatol.

[70] Direito I (2021). Protein aggregation patterns inform about breast cancer response to antiestrogens and reveal the RNA ligase RTCB as mediator of acquired tamoxifen resistance. Cancers (Basel).

[71] Lin X, Chen W, Wei F, Zhou BP, Hung MC, Xie X (2017). Nanoparticle delivery of miR-34a eradicates long-term-cultured breast cancer stem cells via targeting C22ORF28 directly. Theranostics.

[72] Hsieh PL, Liao YW, Hsieh CW, Chen PN, Yu CC (2020). Soy isoflavone genistein impedes cancer stemness and mesenchymal transition in head and neck cancer through activating miR-34a/RTCB axis. Nutrients.

[73] Peach SE, York K, Hesselberth JR (2015). Global analysis of RNA cleavage by 5′-hydroxyl RNA sequencing. Nucleic Acids Res.

[74] Junjappa RP, Patil P, Bhattarai KR, Kim HR, Chae HJ (2018). IRE1alpha implications in endoplasmic reticulum stress-mediated development and pathogenesis of autoimmune diseases. Front Immunol.

[75] Xiang C, Wang Y, Zhang H, Han F (2017). The role of endoplasmic reticulum stress in neurodegenerative disease. Apoptosis.

[76] Madden E, Logue SE, Healy SJ, Manie S, Samali A (2019). The role of the unfolded protein response in cancer progression: from oncogenesis to chemoresistance. Biol Cell.

[77] Logue SE (2018). Inhibition of IRE1 RNase activity modulates the tumor cell secretome and enhances response to chemotherapy. Nat Commun.

[78] Cox AD, Fesik SW, Kimmelman AC, Luo J, Der CJ (2014). Drugging the undruggable RAS: mission possible?. Nat Rev Drug Discov.

[79] Akiyama Y, Takenaka Y, Kasahara T, Abe T, Tomioka Y, Ivanov P (2022). RTCB complex regulates stress-induced tRNA cleavage. Int J Mol Sci.

[80] Tao EW, Cheng WY, Li WL, Yu J, Gao QY (2020). tiRNAs: a novel class of small noncoding RNAs that helps cells respond to stressors and plays roles in cancer progression. J Cell Physiol.

